# Network-constrained spatio-temporal clustering analysis of traffic collisions in Jianghan District of Wuhan, China

**DOI:** 10.1371/journal.pone.0195093

**Published:** 2018-04-19

**Authors:** Yaxin Fan, Xinyan Zhu, Bing She, Wei Guo, Tao Guo

**Affiliations:** 1 State Key Laboratory of Information Engineering in Surveying, Mapping and Remote Sensing, Wuhan University, Wuhan, China; 2 China Data Center, University of Michigan, Ann Arbor, United States of America; 3 Wuhan Digital Engineering Research Institute, Wuhan, China; Beihang University, CHINA

## Abstract

The analysis of traffic collisions is essential for urban safety and the sustainable development of the urban environment. Reducing the road traffic injuries and the financial losses caused by collisions is the most important goal of traffic management. In addition, traffic collisions are a major cause of traffic congestion, which is a serious issue that affects everyone in the society. Therefore, traffic collision analysis is essential for all parties, including drivers, pedestrians, and traffic officers, to understand the road risks at a finer spatio-temporal scale. However, traffic collisions in the urban context are dynamic and complex. Thus, it is important to detect how the collision hotspots evolve over time through spatio-temporal clustering analysis. In addition, traffic collisions are not isolated events in space. The characteristics of the traffic collisions and their surrounding locations also present an influence of the clusters. This work tries to explore the spatio-temporal clustering patterns of traffic collisions by combining a set of network-constrained methods. These methods were tested using the traffic collision data in Jianghan District of Wuhan, China. The results demonstrated that these methods offer different perspectives of the spatio-temporal clustering patterns. The weighted network kernel density estimation provides an intuitive way to incorporate attribute information. The network cross K-function shows that there are varying clustering tendencies between traffic collisions and different types of POIs. The proposed network differential Local Moran’s I and network local indicators of mobility association provide straightforward and quantitative measures of the hotspot changes. This case study shows that these methods could help researchers, practitioners, and policy-makers to better understand the spatio-temporal clustering patterns of traffic collisions.

## Introduction

Streets are one of the most common types of physical networks. Human activities in the urban space, although dynamic in nature, are largely constrained by the street networks [1]. Such constraints are an important force for pushing geographical entities to distribute along streets. Events in the urban space are also more likely to be located on, or near, street segments. Traffic collisions are one of the most common types of such events. The World Health Organization reported that road traffic injuries have become the number one cause of death among the age group 15–29, and an estimated 3% of GDP is lost to road traffic deaths and injuries globally [2]. A sustainable transportation system allows the access needs of individuals to be met safely and consistent with human health [3]. Therefore, ensuring traffic safety is a major goal of local Traffic Management Bureaus for the sustainable urban transportation [4–6]. The analysis of traffic collisions is key to reducing traffic injuries [7]. However, maintaining traffic safety is an extremely complex task that involves drivers, vehicles, pedestrians, cyclists, motorcyclists, and road environment, etc. Measuring the road risks at the segment level is critical for traffic officers for resource allocation and policy-making. Traffic officers could also use the information for more targeted management practices. On the other hand, if drivers and pedestrians are aware of locations and time of collision hotspots on the roads, they are more likely to avoid them or adopt more defensive ways when approaching them. Traffic collision analysis provides approaches to visualize the spatial distribution and patterns of road risks, and quantify the risks through a set of spatial statistics. Therefore, the identification of the spatio-temporal clustering patterns of traffic collisions would help practitioners and policy-makers in better understanding the dynamics of collision hot-spots at a finer spatial scale. In recent years, GIS and spatio-temporal analysis methods have increasingly been used to study the characteristics of traffic collisions. These methods include both exploratory ones such as mapping and geovisualization techniques and confirmatory models from spatial statistics [8].

Recent years have seen a growing interest in applying network-constrained spatial analysis methods to study the characteristics of urban events. These exploratory models can quantitatively measure the spatial patterns and interactions of traffic collisions, using the spatial location and time stamp of the events. However, traffic collisions are not isolated events in space. The semantic information and temporal dimension of traffic collisions are also important for the comprehensive understanding of the spatio-temporal clustering patterns.

The semantic information of traffic collisions, including both the inherent attributes and the environmental factors of the location where the collision happens, is also related to the collisions. The inherent attributes include the types of collisions, financial loss, the cause of collisions, the number of vehicles involved, etc. These attributes are often incorporated into a regression model for confirmatory analysis, but they can also be incorporated into exploratory methods. For example, the inherent attributes of collisions could be integrated into the kernel density estimation method, thus, practitioners can observe the spatial pattern from different perspectives. The environmental factors include the characteristic of the location where the event happens, and also the surrounding locations. The characteristics of the location where the event happens are directly related to the event, including the road type, traffic volumes, and the weather condition. The characteristics of the surrounding locations are mostly the characteristics of the surrounding points of interest (POIs), such as grocery stores, parking lots, and hospitals. These POIs might not directly relate to individual collisions, but their spatial distribution might correlate with spatial distributions of collisions collectively.

The temporal dimension of the traffic collisions could be used to detect the changes in the spatial distributions of events. The collision hotspots might emerge, disappear, or move over time. The resulting changes could be presented by showing the spatial analysis results from different time points, with a common comparable scale. For example, we can use kernel density estimations to see how the spatial distribution changes across different times of the day. Another approach would be to first compute a measure from two time points, and then use a hotspot detection method to analyze the changes quantitatively. In addition, although the analysis can be done between two arbitrary time points or periods, a meaningful organization of time would be more useful for practitioners when implementing regulating policies. This includes the monthly or seasonal cycles, day of the week, and time of the day. Researchers have studied other types of events using a variety of temporal organizations, such as crime patterns [9].

This paper attempts to explore the spatio-temporal clustering patterns of traffic collisions with semantic information. The weighted network kernel density estimation is extended to explicitly incorporate attribute information with normalized weights. The resulting visualization provides practitioners with multiple perspectives of the spatial distribution of traffic collisions with a certain normalized attribute. The network cross K-function is applied to investigate the relationship between traffic collisions and different types of POIs. To investigate the temporal change of collision clustering, this work proposed the network-based differential local Moran’s I and Local Indicators of Mobility Associations (LIMA) [10] by substituting the planar weight matrix with the network weight matrix. The network differential Moran's I method quantifies the clustering of changes on the street network, while the network LIMA measures the degree of local concordance and discordance over time. The remainder of this paper is organized as follows: The next section introduces the relevant works from literature. Section 3 describes the study area. Section 4 discusses the network-constrained methods. Section 5 presents a case study of Jianghan District, Wuhan, China. Section 6 gives the discussions and outlines the future work.

## Sustainable traffic safety and spatio-temporal clustering analysis of traffic collisions

A sustainable transport system must be able to provide different modes of transport to people in a safe way [11]. The 2030 Agenda for Sustainable Development of the United Nations has set a target of halving the total number of deaths and injuries from traffic crashes by 2020 [12]. Researchers have also used traffic safety as indicators for the sustainable transport systems [13, 14]. The goal of sustainable safety is to prevent traffic collisions. Researchers have proposed ways to achieve sustainable traffic safety by investigating new road patterns [15] or new hardware, such as eye mark recorders [16]. Yet the urban mobilities in our daily experiences are dynamic and complex. Achieving traffic safety needs more than transportation engineering measures [17]. Thus, it is key to involve all parties, including drivers, pedestrians, and traffic officers, in the process of achieving better traffic safety. In practice, policy-makers tend to impose various security policies. However, safety policies sometimes encourage riskier behaviors, which expose greater risks to the transportation system [18]. Drivers and pedestrians might not always willing to follow rules [19]. Individuals, in general, have difficulties making decisions regarding modes of transport when safety is considered an objective [11]. The spatial analytics of traffic collisions will provide all parties with a toolset to measure road risks at finer spatio-temporal scale.

The spatial analytic methods for traffic collision analysis can be generally classified into three types [7]: mapping and topological analysis, identification of clustering patterns, and analysis of contributory factors. This work focus on the second type. Since traffic collisions are constrained by the street network, traditional planar spatial methods introduce a systematic bias in the analysis result [20]. The Ripley’s K-function [21], a widely used method to detect spatial aggregation, might possibly over-detect or underestimate the aggregation in network-constrained phenomena [22, 23].

Network-constrained methods have been extensively studied for finer-scale urban analyses in recent years. These methods can be broadly classified into event-based approaches and link-based approaches [24]. The event-based approach includes the network kernel density estimation (KDE) [25], and the K-function [26], and moving-segment approach [27]. In particular, the network KDE and K-function have been applied widely in recent years for exploring the spatial patterns of traffic collisions and other types of events [22, 28–33]. Researchers have then started to incorporate semantic information into the analysis pipeline. Ni et al. [34] proposed a weighted kernel density estimation method for studying the spatial distribution characteristics of healthcare facilities. Rui et al. [35] used the network cross K-function to study the spatial correlation between Suguo hypermarkets with other hypermarkets and commercial centers. Our work built upon the weighted kernel density estimation method with normalized weights to study the spatial patterns of traffic collisions. The network cross K-function is used to investigate the spatial aggregation patterns between traffic collisions and different types of POIs.

The link-based approaches tried to identify the hot spots by using local spatial autocorrelation tests [36, 37]. Yamada and Thill [20] used the local Moran’s I, Local Getis, and Ord G statistics in the local indicators of network-constrained clusters (LINCS) for detecting local-scale clustering of highway accidents. These methods can detect street segments with statistically significant patterns by use of Monte Carlo simulation procedures. Recent years have also seen an increasing interest in using time geography to measure traffic collision risk [38, 39]. In other areas, such as crime pattern analysis, the time dimension has been used in Markov analysis and combined with other methods, such as Moran’s I [40–42]. Researchers have proposed other types of extensions in the network space. Xianrui and Zhongren [43] proposed a spatio-temporal K-function over the network to analyze taxi load-unload data. Eckley and Curtin [44] used the spatio-temporal interaction Knox test on traffic collision data. Other statistical models are often used for analysis of contributory factors, such as spatial lag model [45], Bayes models [46–48], and Geographically weighted regression [49–51]. This work extended the differential Local Moran’s I and LIMA into the network space, which provides a set of new local measures to quantify the spatio-temporal clustering at the road segment level.

## Study area and data

Wuhan is a rapidly growing city in central China and the capital of Hubei province. According to the Statistical Communiqué of Wuhan on the 2016 National Economic and Social Development [52], the number of permanent residents was 10,766,200 people at the end of 2016. The number of cars has reached 2.31 million, and there are 489 bus routes in operation. Jianghan District is one of the seven major urban districts in Wuhan. It is also the most densely populated, while also the most prosperous, district. Jianghan District has a total area of 33.43 square kilometers and lies on the north shore of the Yangtze River. The data used in this work include traffic collisions, POIs, and the road network. All these data were fully anonymized before we accessed them.

**Fig 1:**
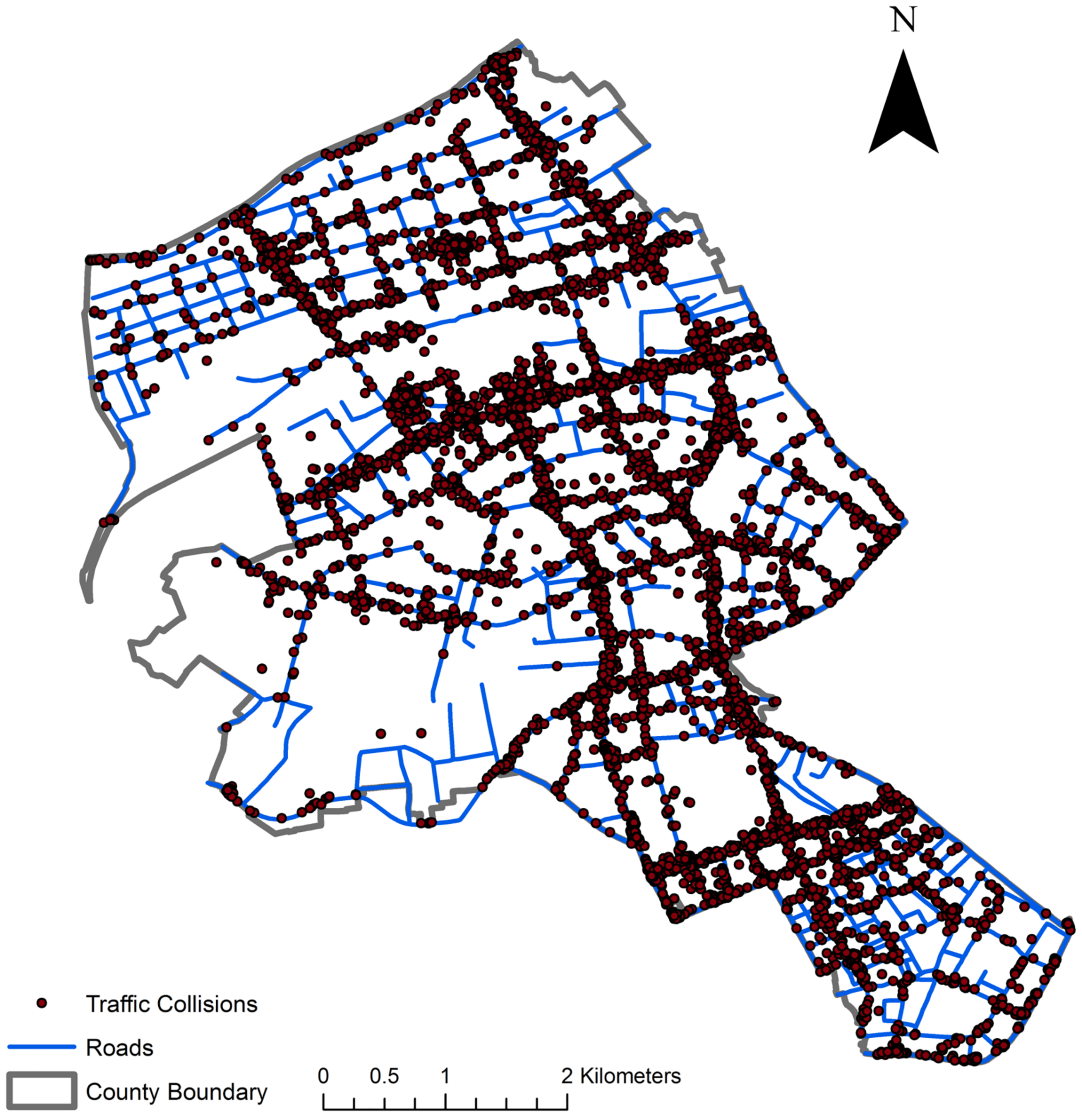
Traffic collision distribution in Jianghan District, Wuhan, China.

The Wuhan Traffic Management Bureau started to equip traffic police with personal data assistants from mid-2016. The traffic collision data collected after that time are better in quality and coverage. This work uses the traffic collision data in Jianghan District from 1 July 2016 to 31 December 2016. There are a total of 11,445 collisions with locations recorded during this period. Fig 1 displays the overall distribution of these events with the street network. It shows a clear clustering tendency of the traffic collisions in certain road segments.

**Fig 2:**
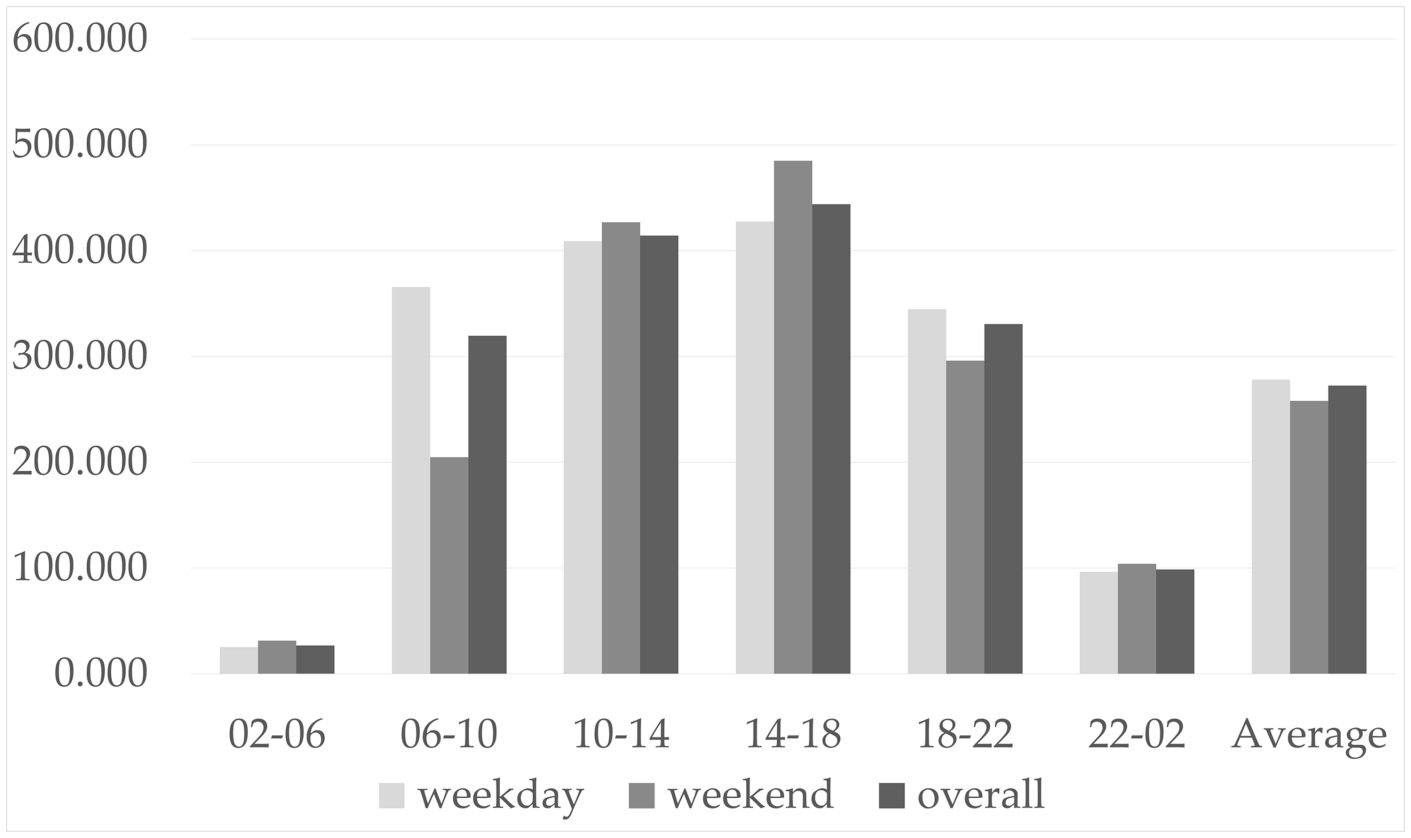
Traffic collision statistics by the hour of the day and weekday/weekends.

Fig 2 shows the number of collisions under two temporal organizations: the hour of the day, and weekday/weekend. The horizontal axis represents the hour of the day is categorized into six periods, plus an extra period that represented the average count. The vertical axis represents the daily average of the cumulative count by weekdays, weekends, and overall, respectively. Unexpectedly, the collisions are mostly aggregated in the daytime. It also suggested that although no significant difference exists between weekdays and weekends, overall, their structure does differ when taking into account the hour of the day factor. This is particularly evident in the morning (6–10). Table 1 illustrates the count of collisions by the ranges of the direct financial loss. Most of the collisions are minor incidents and do not incur any direct financial losses.

**Table 1:** The count of collisions by the ranges of direct financial loss.

Direct financial loss (Yuan)	Count
0	10,616
1–100	50
101–500	46
501–1,000	665
1,001–5,000	64
5,000+	4

**Fig 3:**
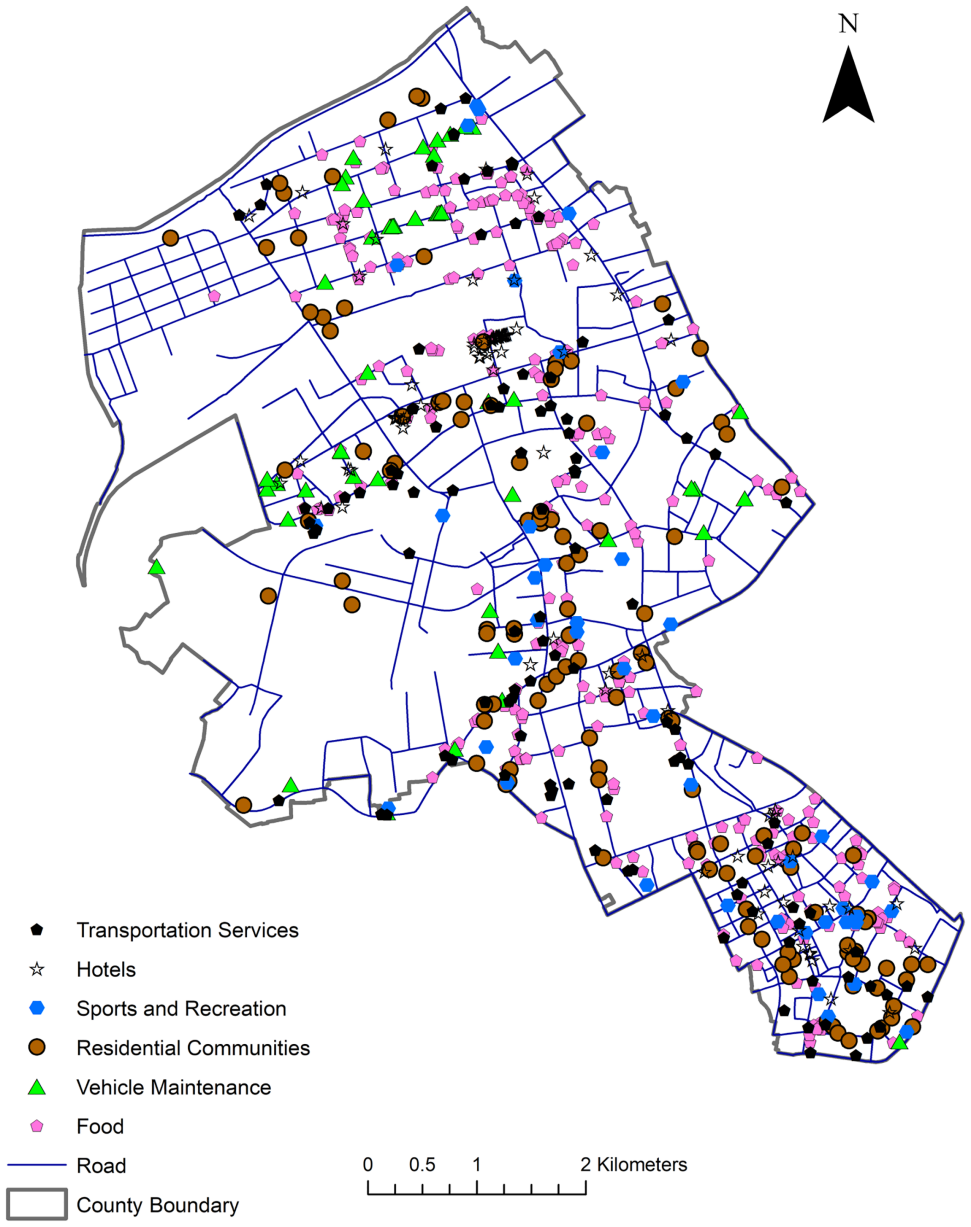
POI distribution in Jianghan District, Wuhan, China.

Six types of POIs are integrated into this study for network cross K-function analysis. The counts for each type of POI points are given in Table 2. The Transportation Services refer to parking lots and public transit stations. The overall spatial distribution of these POI points is displayed in Fig 3.

**Table 2:** The count of POIs by types in Jianghan District, Wuhan, China.

POI Type	Count
Transportation Services	107
Hotels	87
Sports and Recreation	52
Residential Communities	127
Vehicle Maintenance	49
Food	280

## Network-constrained analysis

The street network in this work is formulated as an undirected and planar network N = (V, L), with a set of nodes V and edges L. The event points E = {*e*_1_, …, *e*_*n*_} refers to the traffic collisions. Each event point *e* is represented by its location, time, and a set of attributes. The event points in E are assumed to follow a probabilistic point process that distributed on the network N. The other type of points are the POI points P = {*p*_1_, …, *p*_*m*_}, and this work represent each POI point by its location and type. For simplicity, P is assumed to be a fixed set of points snapped to the network.

### Weighted network kernel density estimation

The network-constrained KDE is a direct extension of the planar KDE into the network space. The core idea is to divide the network space into linear pixels [25] and uses the shortest path between event points to calculate the distance, instead of using Euclidian distance. For an arbitrary point q on N, the weighted kernel estimator is represented as:

$$\lambda \left(q\right)=\frac{1}{n}\sum_{i=1}^{n}w_{i}*K_{i}\left(q\right)$$ (1)

where *w*_*i*_ is the weight for the event point *i*. This work used a quantile mapping method to map an original event attribute into a weighted vector. This is derived from [53], which computes the weights of links for network Voronoi diagrams. The weight vector falls into a normalization interval R = [*r*_*min*_, *r*_*max*_], a user-defined parameter that controls the influence of the weights on the kernel estimator. Suppose the original event attribute vector is c. The process starts with the sorting of c, and then the ranks of values are used to compute the normalized value. The number of distinct ranks is *n*_*d*_ = *n* − *n*_*e*_, where *n*_*e*_ is the number of equal values in c. The event with the kth largest value will be normalized to:

$$c^{\prime }=\frac{\left(k-1)*(r_{max}-r_{\text{min}}\right)}{\left(n_{d}-1\right)}+r_{\text{min}}$$ (2)

where *K*_*i*_(*q*) is the kernel function at the kernel center i. The equal-split kernel function is used to prevent biased estimates at road intersections [54]. Suppose the shortest path from *i* to *q* contains *p* nodes: *v*_1_, …, *v*_*p*_, and let *n*_*i*_ represent the degree of the node *v*_*i*_. *K*_*i*_(*q*) is given by:

$$K_{i}\left(q\right)=\{\begin{array}{cc} \frac{k\left(d\left(q,i\right),h\right)}{\left(n_{1}-1\right)\left(n_{2}-1\right)\ldots \left(n_{p}-1\right)} & for\;0\leq d\left(q,i\right)\leq h\\ 0 & for\;d\left(q,i\right)>h \end{array}$$ (3)

where *h* is the bandwidth, while *k*(*d*(*q*, *i*), *h*) is the base kernel function. The idea is to divide the kernel values at each node along the path from i to q, and distribute them to adjacent edges. It is accepted by the research community that the form of the kernel function is less important than the choice of bandwidth [55, 56]. The basic units of computation in the network KDE relies on lixels, and the density values are output for each lixel [33]. The lixel length determines the computational intensity.

### Network cross K-function

The K-function method is considered as an approach to investigate the second-order characteristics of a spatial point process, which is widely used to measure spatial dependence. The network cross K-function extends the measure to take into account two different types of points [35]. In other words, the cross K-function quantifies the spatial interrelationships between two types of point sets. The theoretical form of the cross K-function could be written in the following form:

$$K_{ba}(d)=\frac{1}{\rho_{b}}\frac{\sum_{i=1}^{n}n(d,b|a_{i})}{n}$$ (4)

where *ρ*_*b*_ is the density of point type *b* on the network, while *n*(*t*, *b*|*a*_*i*_) is the number of points of type *b* that are within distance *d* from point *i* of type *a*. The distance is also calculated by the shortest path method. In the context of this work, the traffic collisions are the type-*b* points, and POI points are the type-*a* points. *K*_*ba*_(*d*) could be written as:

$$K_{ba}(d)=\frac{1}{\rho_{b}}\frac{\sum_{i}\sum_{j}I(|s(a_{i},b_{j})|<d)}{n}$$ (5)

where |*s*(*a*_*i*_, *b*_*j*_)| denote the distance of shortest path from POI *a*_*i*_ to the traffic collision *b*_*j*_, and *I*(|*s*(*a*_*i*_, *b*_*j*_)| < *d*) is the indicator function with the value 1 if the distance is smaller than *d* and 0 otherwise. Okabe also proposed a transformation method to transform a non-uniform network into a uniform network [57]. Similar to the planar K-function, the Monte Carlo simulation method is used to test the distribution pattern of point events. This can be done by generating simulated point patterns on the network repeatedly according to the completely spatial random assumption. Then, the observed K-function curve is compared with the simulated K-function curve. Judging from the relations of the curves, we can then tell whether the traffic collisions are clustered around, dispersed from, or unrelated to certain types of POIs.

### Network differential local Moran’s I method

The local Moran’s I statistic developed by Anselin [58] is a widely accepted measure of spatial autocorrelation. For region *i*, the local I statistic for an attribute v is defined as:

$$I_{i}=z_{i}\sum_{j}w_{ij}z_{j}$$ (6)

where *z*_*i*_ and *z*_*j*_ are the normalized value of v, and *w*_*ij*_ is a binary indicator of whether areas i and j are adjacent. This adjacency relationship is represented as the spatial weight matrix W. Positive values of the local Moran’s I statistic suggests a clustering tendency, while negative values indicate spatial dispersiveness of the distribution.

The differential local Moran’s I method is a natural extension of the Moran’s I statistics. It measures the spatial patterns of the changes of the same attribute between two different times [59]. The form of the differential local Moran’s I is as follows:

$$I_{i}=z\left(v_{it_{2}}-v_{it_{1}}\right)\sum_{j}w_{ij}z\left(v_{jt_{2}}-v_{jt_{1}}\right)$$ (7)

where $z(v_{it_{2}}-v_{it_{1}})$ and $z(v_{jt_{2}}-v_{jt_{1}})$ are the normalized values of the changes in v from time *t*_1_ to *t*_2_. The simulation process is done by using conditional random permutations. In some circumstances where the analyst wants to compare two time periods, the attribute could be normalized by the length of two periods, which gives the following:

$$I_{i}=z\left(\frac{v_{it_{2}}}{L_{t_{2}}}-\frac{v_{it_{1}}}{L_{t_{1}}}\right)\sum_{j}w_{ij}z\left(\frac{v_{jt_{2}}}{L_{t_{2}}}-\frac{v_{jt_{1}}}{L_{t_{1}}}\right)$$ (8)

where $L_{t_{2}}$ and $L_{t_{1}}$ are the length of the two periods. For example, if we would like to compare the event distribution between weekdays and weekends, the length of the periods could be measured in days. Then $L_{t_{2}}$ and $L_{t_{1}}$ would be 5 and 2, respectively. In this work, we use the number of traffic collisions as the attribute v. The periods used in the analysis are of a certain temporal organization, such as weekday/weekend, or the hour of the day.

This work extends the differential Moran’s I into the network space by substituting the planar weight matrix *W* with the network weight matrix *W*_*N*_. Therefore, *w*_*i*,*j*_ defines the neighboring relationships between two network segments. Researchers have previously used *W*_*N*_ to compute the local Moran’s I, Local Getis, and Ord G statistics in the network space [20, 32]. Two types of network weight matrices exist: the node-based matrix and the distance-based matrix. The node-based matrix will only treat network segments as neighbors when they are directly connected. The distance-based matrix determines the neighboring relationships based on whether the distance between the centers of two segments is less than a distance threshold or not. We adopted the distance-based matrix because it can represent the segment relationships more flexibly by controlling the threshold parameter and it is commonly used in the literature [20, 33].

### Network local indicators of mobility association

The local indicators of mobility association measures are derived from the global indicators of mobility association, which is further derived from the general rank correlation coefficient proposed by Kendall [60]. Here we consider the two observation vectors $v_{t_{1}}$ and $v_{t_{2}}$ that represent the same variable in two periods. The coefficient $\tau (v_{t_{1}},v_{t_{2}})$ is given by:

$$\tau (v_{t_{1}},v_{t_{2}})=\frac{\sum_{i=1}^{n-1}\sum_{j=i+1}^{n}sgn(v_{it_{1}}-v_{jt_{1}})sgn(v_{it_{2}}-v_{jt_{2}})}{n(n-1)/2}=\frac{C-D}{n(n-1)/2}$$ (9)

where the *sgn* function extracts the sign of the difference between two units, thus taking values 1 or –1. If $sgn(v_{it_{1}}-v_{jt_{1}})sgn(v_{it_{2}}-v_{jt_{2}})=1$, the pair of observation between unit *i* and *j* is concordant across two periods *t*_1_ and *t*_2_. If $sgn(v_{it_{1}}-v_{jt_{1}})sgn(v_{it_{2}}-v_{jt_{2}})=-1$, the pair is disconcordant. C and D represent the number of concordant and disconcordant pairs. The use of ranks makes Kendall’s *τ* robust to departure from bivariate normality [61].

To consider ties in the observations which would lead $sgn(v_{it_{1}}-v_{jt_{1}})sgn(v_{it_{2}}-v_{jt_{2}})=0$, extra pairs could be accounted for in the denominator [61], which gives:

$$\tau \prime (v_{t_{1}},v_{t_{2}})=\frac{C-D}{\sqrt{C+D+E_{t_{1}}}\sqrt{C+D+E_{t_{2}}}}$$ (10)

where $E_{t_{1}}$ represents the number of extra pairs introduced when $sgn(v_{it_{1}}-v_{jt_{1}})\neq 0$ and $sgn(v_{it_{2}}-v_{jt_{2}})=0$, while $E_{t_{2}}$ represents the number of extra pairs introduced when $sgn(v_{it_{1}}-v_{jt_{1}})=0$ and $sgn(v_{it_{2}}-v_{jt_{2}})\neq 0$. The values of *τ′* falls on the range [–1,1]. A value of 1 indicates that all pairs are concordant. This means that larger values of *τ′* implies less distributional mixing from period *t*_1_ to *t*_2_. Detailed explanations for handling ties are given in [60].

Rey proposed a spatial concordance measure based on Kendall’s *τ* measure [62]. The spatial measure is based on the decomposition of the pairs of observation into those that are neighbors and those that are not. Suppose a binary spatial weight matrix W is constructed to represent whether units *i* and *j* are neighbors, define matrix $\overset{-}{W}=J-W-I$, where *J* is a matrix of ones and *I* is an identity matrix. The measure *τ* can thus be decomposed into:

$$\tau (v_{t_{1}},v_{t_{2}})=\psi \tau_{w}(v_{t_{1}},v_{t_{2}})+(1-\psi )\tau_{\overset{-}{w}}(v_{t_{1}},v_{t_{2}})$$ (11)

where *ψ* = ∑_*i*_∑_*j*_*w*_*i*,*j*_/*n*(*n* − 1), and *τ*_*w*_ and $\tau_{\overset{-}{w}}$ are the decomposed concordance measures for the neighboring pairs and the non-neighboring pairs. *τ*_*w*_ is then considered as a type of Global Indicators of Mobility Association (GIMA), given by:

$$\tau_{w}(v_{t_{1}},v_{t_{2}})=\frac{\sum_{i}\sum_{j}w_{i,j}sgn(v_{it_{1}}-v_{jt_{1}})sgn(v_{it_{2}}-v_{jt_{2}})}{\sum_{i}\sum_{j}w_{i,j}}$$ (12)

The approach of handling ties in Eq (10) could be used to incorporate extra pairs in computing *τ*_*w*_. Rey further constructs three types of Local Indicators of Mobility Association (LIMA) [10]: the local concordance *τ*_*i*_, the neighbor set LIMA ${\tilde{\tau }}_{i}$, and the neighborhood set LIMA ${\tilde{\tilde{\tau }}}_{i}$. Let $concordance(i,j)=sgn(v_{it_{1}}-v_{jt_{1}})sgn(v_{it_{2}}-v_{jt_{2}})$, then be:

$$\tau_{i}=\frac{\sum_{j\neq i}concordance(i,j)}{n-1}$$ (13)

$${\tilde{\tau }}_{i}=\frac{\sum_{j}w_{i,j}concordance(i,j)}{\sum_{j}w_{i,j}}$$ (14)

$${\tilde{\tilde{\tau }}}_{i}=\frac{\sum_{m\in {NS}_{i}}\sum_{n\in {NS}_{i},n\neq i}concordance(m,n)}{\left|{NS}_{i}|(|{NS}_{i}|-1\right)}$$ (15)

where *NS*_*i*_ is the neighborhood set of *i* plus *i*. This work will consider ${\tilde{\tau }}_{i}$ and ${\tilde{\tilde{\tau }}}_{i}$ in the case study which takes the local spatial context into account. The ${\tilde{\tau }}_{i}$ measure investigate the local concordance between a unit and its neighbors, while ${\tilde{\tilde{\tau }}}_{i}$ extends ${\tilde{\tau }}_{i}$ by conducting the computations between all pairs of observations in a unit’s neighborhood set. The inference is done by using conditional random permutations.

Similar to the network differential local Moran’s I, this work extends the LIMA measures into the network space by using the network weight matrix *W*_*N*_. The LIMA measures use binary weight matrices, thus *w*_*i*,*j*_ defines whether two network segments are neighbors, while *NS*_*i*_ is the neighboring segments of the segment *i*.

### Network computations

**Fig 4:**
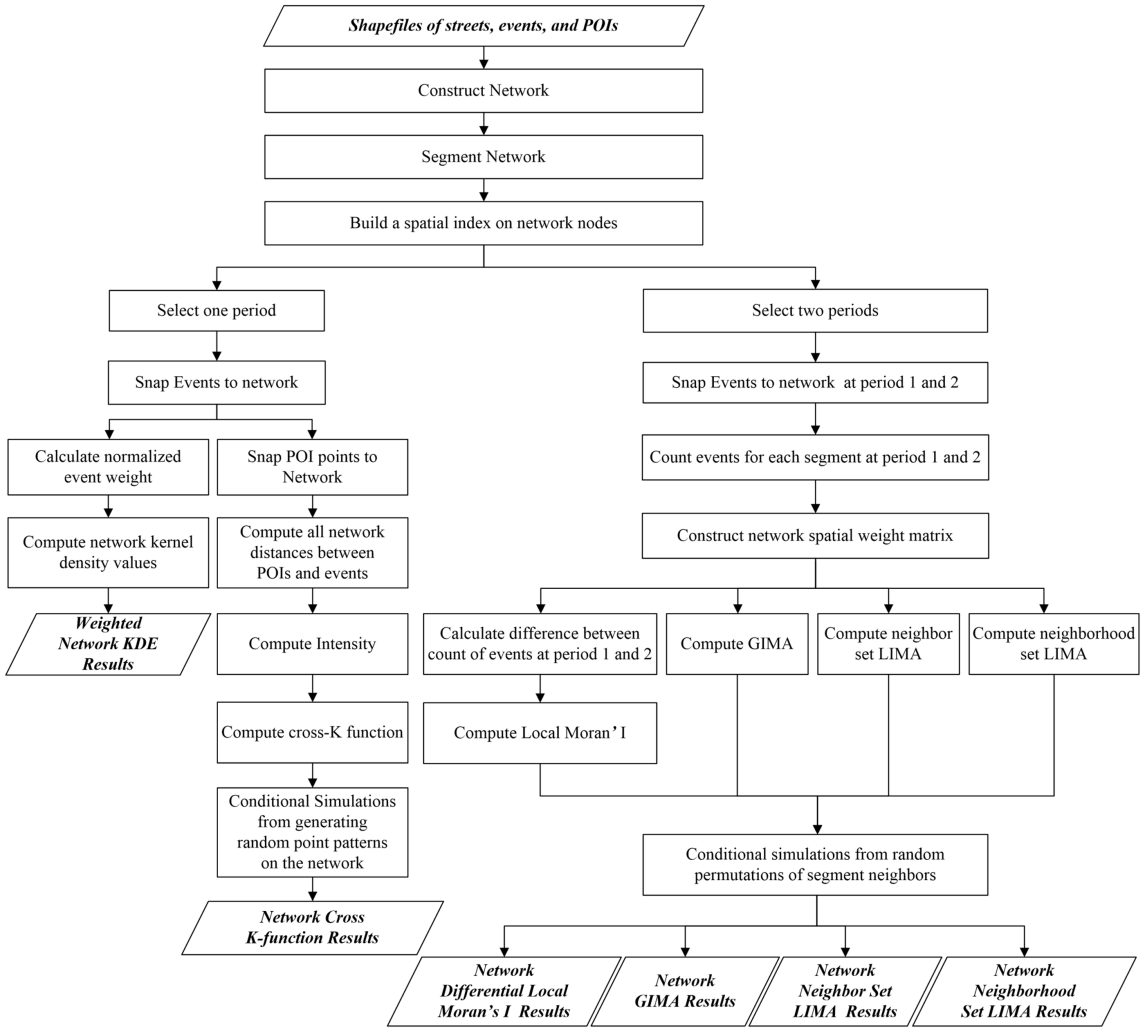
The workflow of network-constrained analysis.

The input to the network-constrained analysis methods are the shapefiles of the streets, events, and POIs. Fig 4 gives a synthesized workflow of these methods. During the preprocessing phase, the street network is first constructed from the streets and segmented into network segments. It is a common practice to split the network edges into equal sizes approximately [20, 25]. The segment size is predefined by analysts. The street network used in this work is a generalization of the real-world roads, which do not consider lanes and complex intersection structures. This would produce an offset between the event points and the street network. The offset is also subject to errors in GPS readings when recording the events. Therefore, the event points need to be projected into the network. The snapping process of event points and POIs is for finding the nearest edge for an event or POI point. This process can be accelerated by first constructing a spatial index (e.g., R tree) for the network N. For the network KDE and cross K-function analysis, the events points are inserted as endpoints in N. This insertion process will transform N into a new network *N*′ with its original segments split by events points. For the network differential Moran’ I and GIMA/LIMA analysis, the numbers of events on each edge are counted for computing the indicators.

The distance computation on the network is a basic operation for all the analysis methods in this work, but in slightly different ways. The weighted network KDE method finds the distances of other events to an event point within a given bandwidth. The network cross K-function method computes all network distances between all POIs and all events. For the network differential Moran’ I and GIMA/LIMA analysis, a distance-based network spatial weight matrix is constructed based on the distances between network segments. The simulations are done by random permutations of segment neighbors.

## Results

The computations of the methods were implemented in Python. PySAL is leveraged for Local Moran’s I and network computations [63]. The network local indicators of mobility association measures are implemented based on the PySAL-giddy package, which originates from the spatial dynamic module of PySAL for analyzing the dynamics of longitudinal spatial data [64]. The case studies are conducted on a machine with an i7 Intel CPU and 16 GB DDR3 memory.

### Weighted network KDE analysis

**Fig 5:**
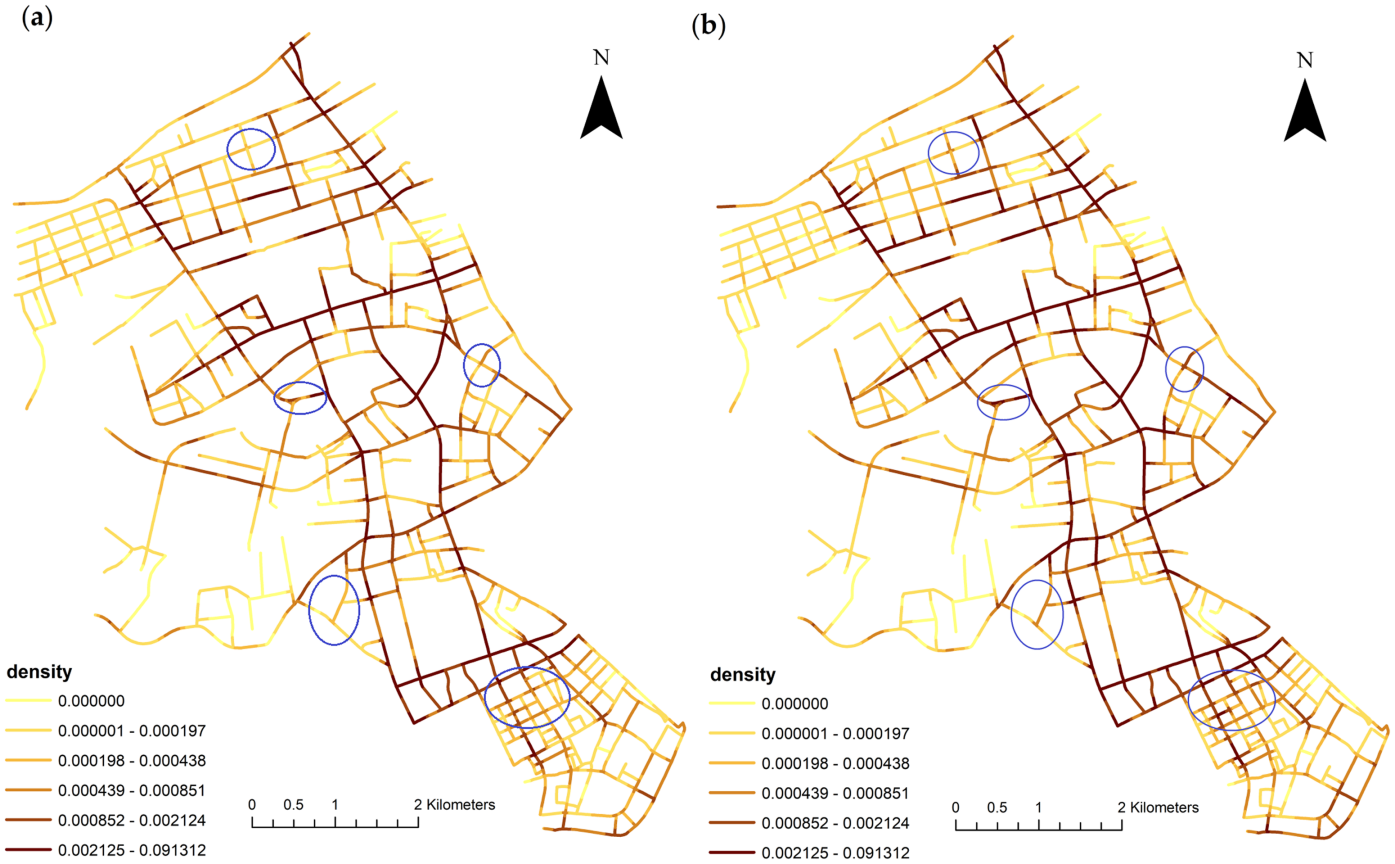
Comparison of unweighted and weighted network KDE for all traffic collisions in Jianghan, Wuhan, China. (**a**) Unweighted KDE; and (**b**) weighted KDE.

The bandwidth parameter is an important issue in the network KDE analysis. Porta et al. [65] proposed a 100–300 m bandwidth in urban applications. This work chose a bandwidth of 200 meters. The length of lixel is set as 40 meters, as suggested in Xie and Yan [25]. The normalization interval for the weighted attribute of direct financial loss is set to [1, 10]. Fig 5 compares the distribution of the unweighted and weighted KDE for all traffic collisions in the experimental data. The blue oval-shaped markers indicate some clear differences and they appear mostly in road intersections.

**Fig 6:**
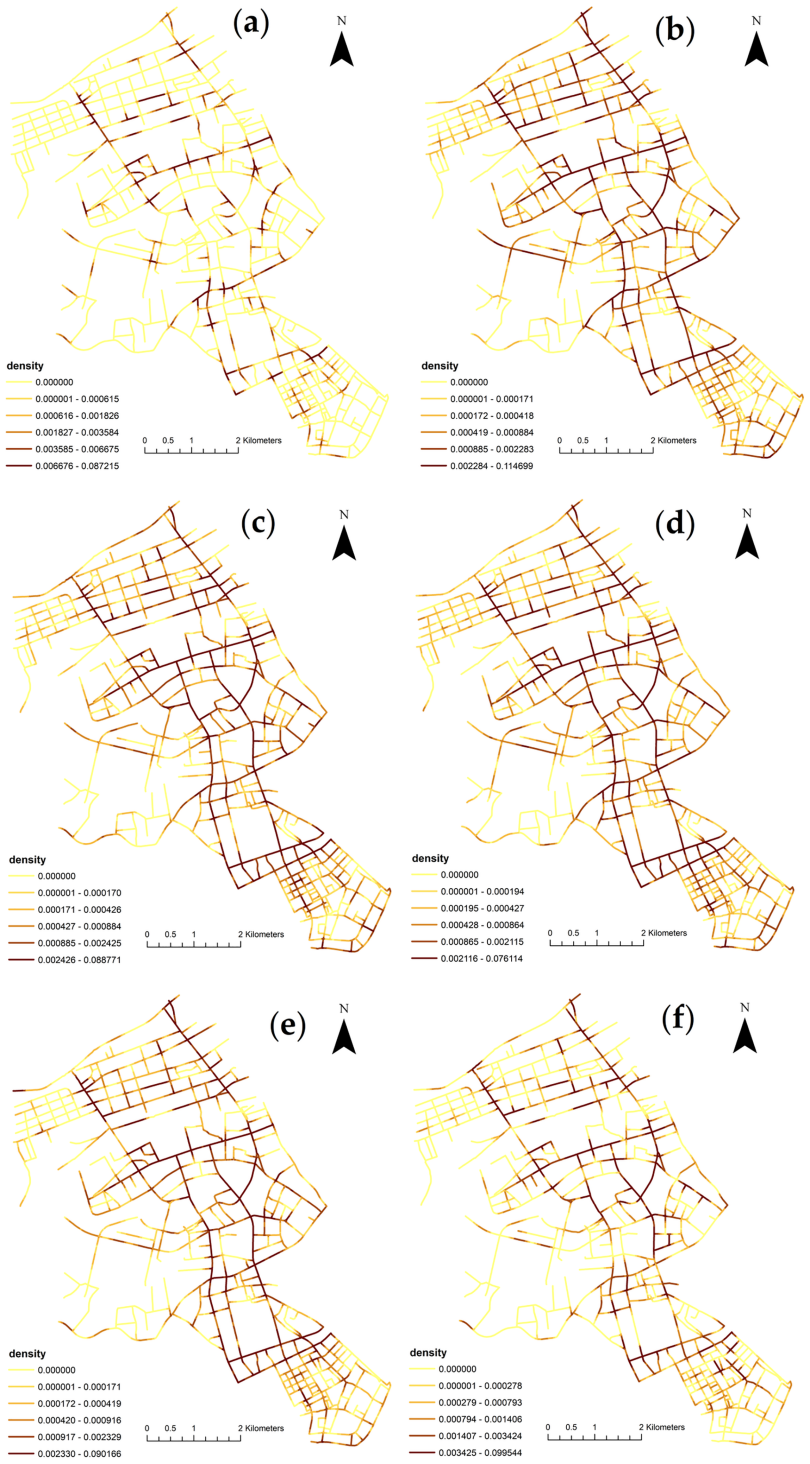
Comparison of weighted network KDE of traffic collisions by the hour of the day in Jianghan, Wuhan, China: (a) 2–6; (b) 6–10; (c) 10–14; (d) 14–18; (e) 18–22; and (f) 22–2.

Fig 6 displays the comparison of six weighted network KDE results of traffic collisions by the hour of the day. The map classifications are all completed using the quantile method. The Figs 5 and 6 clearly show that the spatial structures of traffic collisions are quite different between daytime and nighttime. Traffic collisions on the main road have a relatively higher frequency at all times in a day. During 2–6 in the morning, there are fewer accidents because there are fewer cars, and most of these accidents are distributed around road intersections and main roads. The Jianghan district is the major economic and business center of Wuhan, the traffic flow stays relatively high from 6:00 to 22:00. Most accidents are minor incidents caused by traffic violations such as overtaking, failing to yield, and cut-in. The spatial distribution of traffic collisions remains largely stable from 6:00 to 22:00, while small variations do exist.

### Network cross K-function analysis

**Fig 7:**
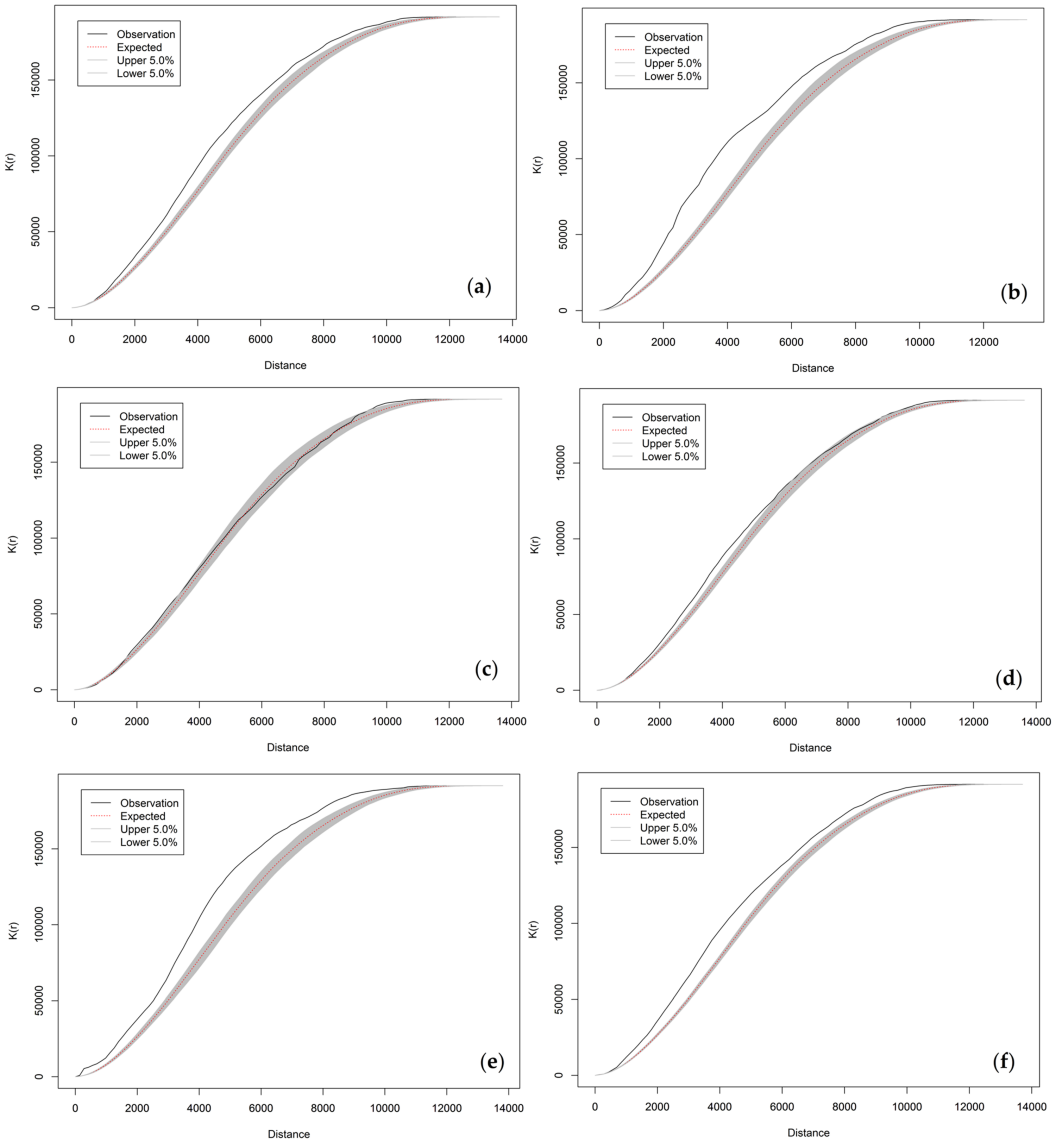
Network cross K-function analysis between traffic collisions and different types of POIs in Jianghan, Wuhan, China: (a) Transportation Services; (b) Hotels; (c) Sports and Recreation; (d) Residential Communities; (e) Vehicle Maintenance; and (f) Food.

The network cross K-function is used to analyze the relationships between traffic collisions and different types of POIs. The results are used to measure quantitatively the degree of network aggregation between traffic collisions and surrounding POIs. The results were plotted in R with outputs from the Python program, and shown in Fig 7. The plots show clearly the relationships between traffic collisions and POIs varies considerably for different POI types.

Among all the POI types, traffic collisions show the strongest degree of network aggregation around POI points of Hotels and Vehicle Maintenance. However, they tend to follow a random distribution around POI points of Sports and Recreation. Mild network aggregation exists for POI points of Transportation Services, Residential Communities, and Food. It is possible to form assumptions of the network aggregations. For example, the aggregation of traffic collisions around Vehicle Maintenance POIs might be due to the fact that the vehicles going to these POIs might have issues at that time. The collisions around hotels might be due to several factors, including a large volume of incoming and outgoing traffic, driving under the influence, and other traffic violations such as overtaking and cut-in. The mild aggregation of collisions around Transportation Services, Residential Communities, and Food might be largely because of scratch incidents related to parking violations. The Sports and Recreation POIs mostly have direct access to public transportation and less traffic flow, and thus there is no significant network aggregation of traffic collisions around them. However, it is important to note that the network cross K-function analysis is still a descriptive measure of the network aggregation. Therefore, it is more suitable to be used in the exploratory phase. The assumptions formed in this phase needs to be validated in further statistical regression analysis with supplementary data.

### Network differential local Moran’s I analysis

**Fig 8:**
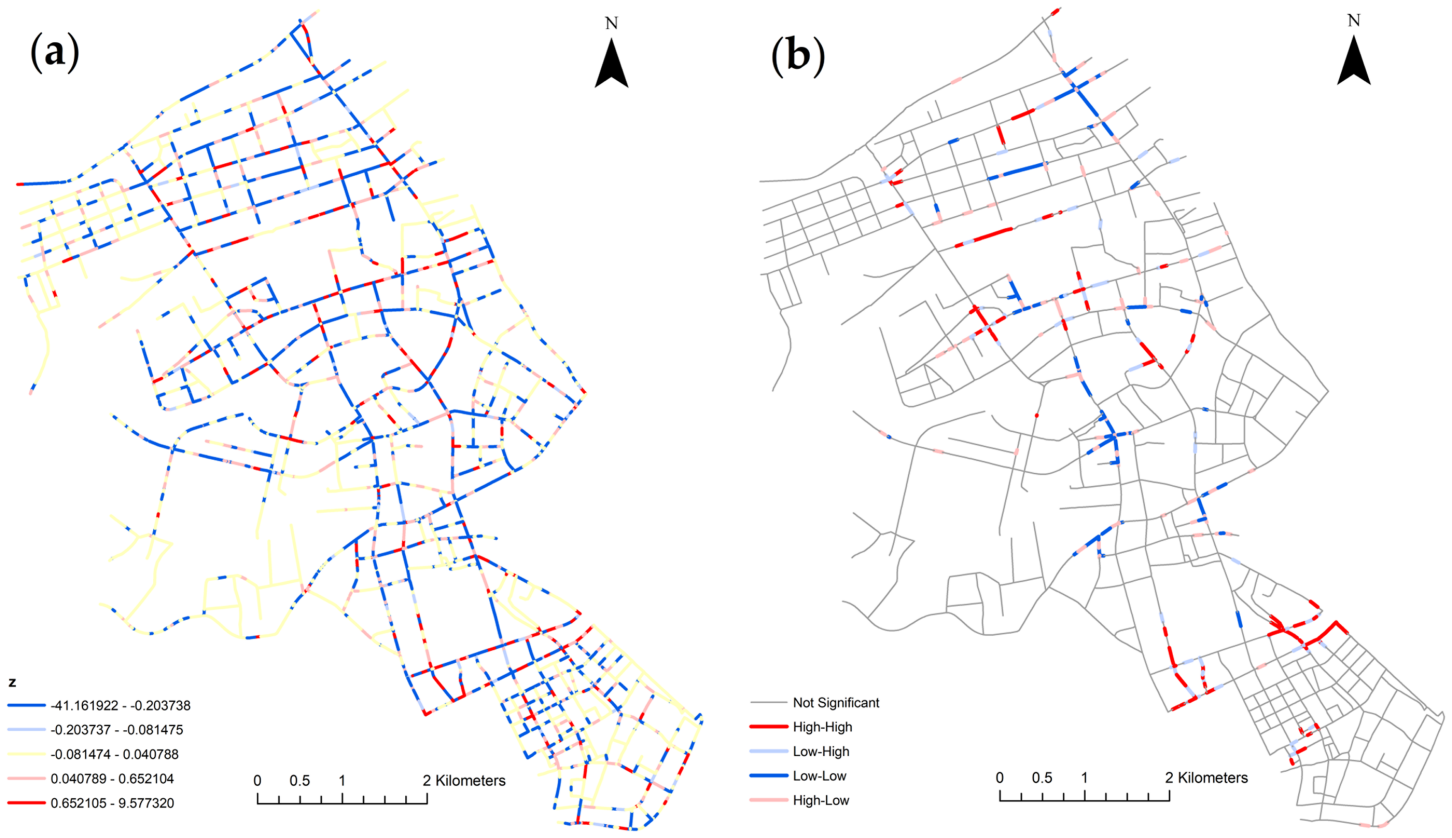
The network differential local Moran’s I analysis from weekdays to weekends in Jianghan, Wuhan, China: (a) Z values; and (b) pattern maps.

The network differential Local Moran’s I method is used to quantify the changes in space between two periods. The segmentation width is set to 100 meters in this analysis as this is the standard distance in management practice for segmenting roads in the Wuhan Traffic Management Bureau. The bandwidth threshold for computing the spatial weight matrix is set to 300 meters. This means that a road segment will be neighbors to all segments that are within 300 meters in network distance. The significance level is set to 0.05 and the number of iterations in the Monte Carlo simulation is set to 999 times. In this study, we first grouped all collisions into weekdays and weekends and compared these two periods. Fig 8 shows the Z value distributions and patterns of the changes from weekdays to weekends. The Z value distribution map indicates the normalized values of changes. The negative values suggest a drop in the number of traffic collisions from weekdays to weekends, while positive values suggest an increase in the number. The pattern map gives a clear view of where the cluster segments locate. The patterns correspond to the changes in the number of traffic collisions from weekdays to weekends. Therefore, it does not directly reflect the large or small number of traffic incidents on road segments. Particularly, the high-high segments indicate clusters with high and significant increases in the number of traffic incidents. These places are mostly around large commercial areas where citizens frequently go on the weekends. Oppositely, the low-low segments indicate clusters with large and significant drops in the number of traffic incidents. Many of these places are near residential communities and industrial areas with less traffic flow on the weekends.

**Fig 9:**
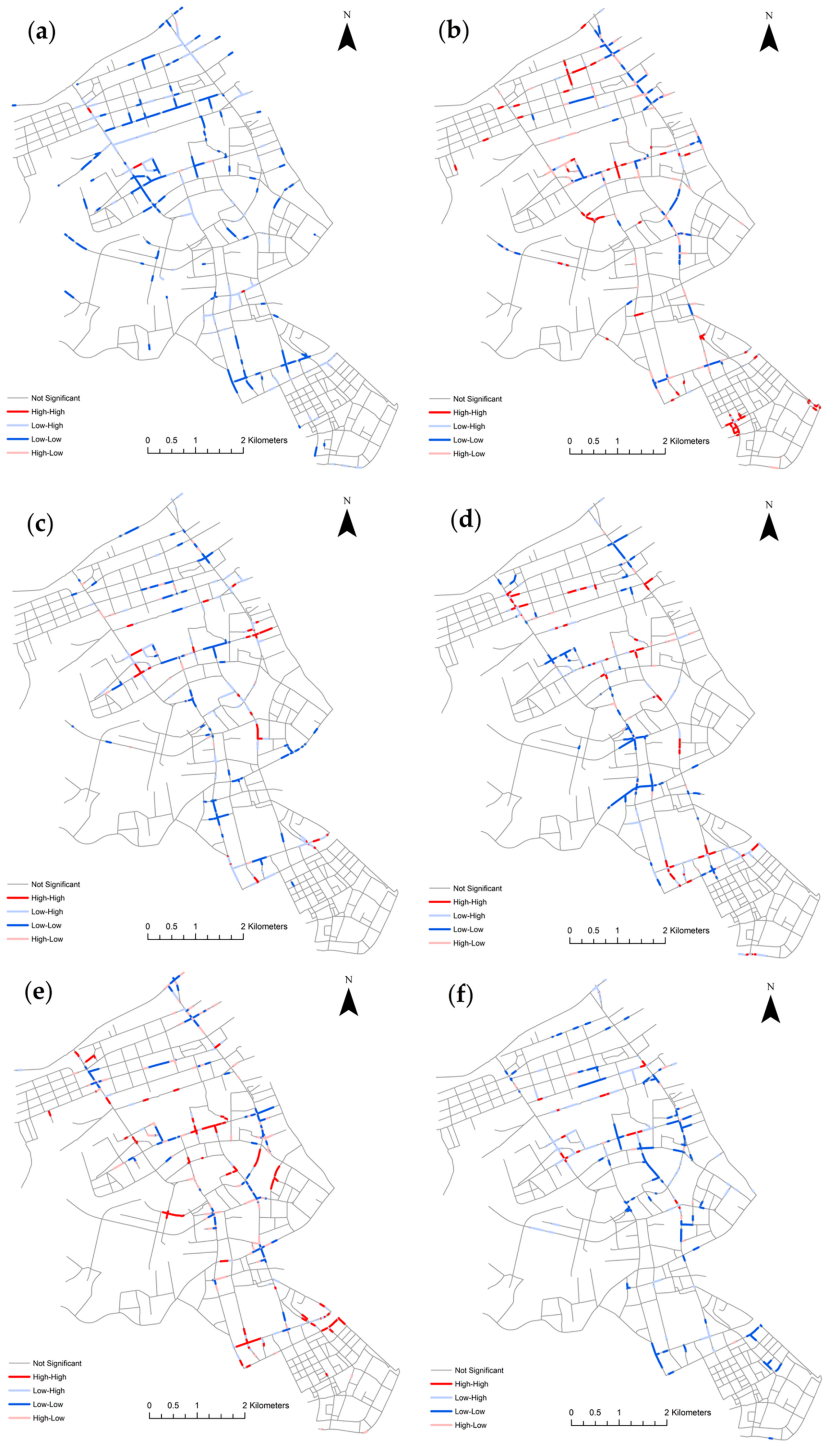
Network differential Local Moran’s I pattern maps of the changes of six hours of day periods from weekdays to weekends in Jianghan, Wuhan, China: (a) 2–6; (b) 6–10; (c) 10–14; (d) 14–18; (e) 18–22; and (f) 22–2.

To demonstrate the use of finer temporal organizations, we first grouped the events by weekdays and weekends, then under each group, classified the events further by the hour of the day. Then the two same hours of the day periods from weekdays and weekends can be compared. Fig 9 shows the patterns of the changes of these six hours of the day periods from weekdays to weekends. During the 2–6 and 22–2 periods, the low-low segment clusters show that there are significant drops in the number of collisions from weekdays to weekends. This is because collisions on weekdays spread across the whole area, while collisions on weekends are more concentrated in certain areas. Starting at 6:00 AM, some road segments emerge as high-high clusters. This indicates that relatively more collisions happen on those clustered segments on weekends than weekdays, even though the total number of collisions on weekdays is larger than weekends. The results indicate that temporal analysis alone (as in Table 2) cannot capture the spatial dynamics of traffic collisions. The differential Local Moran’s I provides an effective tool to quantify and map the micro-level change of collisions in the spatial dimension.

### Network LIMA

The network LIMA analysis offers another angle of the spatio-temporal clustering of traffic collisions. The neighbor set LIMA ${\tilde{\tau }}_{i}$ investigates the concordance relationship between a focal segment and its neighbors. The neighborhood set LIMA ${\tilde{\tilde{\tau }}}_{i}$ expands ${\tilde{\tau }}_{i}$ by taking all pairs of segments belonging to the neighborhood set of a segment into the computation. As the LIMA statistics are based on the ranks of the variables, there is a potential loss of statistical power. However, they are robust to outliers and have better generality by relaxation of the underlying correlation statistics [10]. Thus, the network differential Local Moran’s I Analysis and network LIMA are complementary to each other. The network differential Local Moran’s I Analysis detects the significant clusters of changes, while the network LIMA detects segments with significant rank changes relative to its neighbors or segments with significant rank changes among its neighbors.

In this work, we use the network LIMA to quantify the spatio-temporal patterns of rank concordance of the traffic collisions between six hours of day periods. In accordance with the network differential Local Moran’s I analysis, the segmentation width is set to 100 meters, and the bandwidth threshold for computing the spatial weight matrix is set to 300 meters. The GIMA is used first to investigate the global concordance between the six hours of day periods. Table 3 displays the symmetric concordance matrix, with significant values (*p* < 0.05) indicated in bold and with asterisks. The matrix shows that the period 2–6 has the largest rank changes with all other periods, but the *τ*_*w*_ values are not significant, possibly because that the majority of segments have no collisions during late at night. A similar trend exists for the period 22–2. The only significant changes happen between the pairs of 6–10 and 10–14, 6–10 and 18–22, as well as 10–14 and 18–22. Overall, the positive values in the matrix shows that concordance is the dominant pattern.

**Table 3:** The matrix of global concordance (*τ*_*w*_) between the six hours of day periods.

	2–6	6–10	10–14	14–18	18–22	22–2
2–6	-	0.232	0.233	0.201	0.251	0.235
6–10	0.232	-	0.46*	0.464	0.467*	0.355
10–14	0.233	0.46*	-	0.487	0.504*	0.344
14–18	0.201	0.464	0.487	-	0.496	0.337
18–22	0.251	0.467*	0.504*	0.496	-	0.385
22–2	0.235	0.355	0.344	0.337	0.385	-

**Fig 10:**
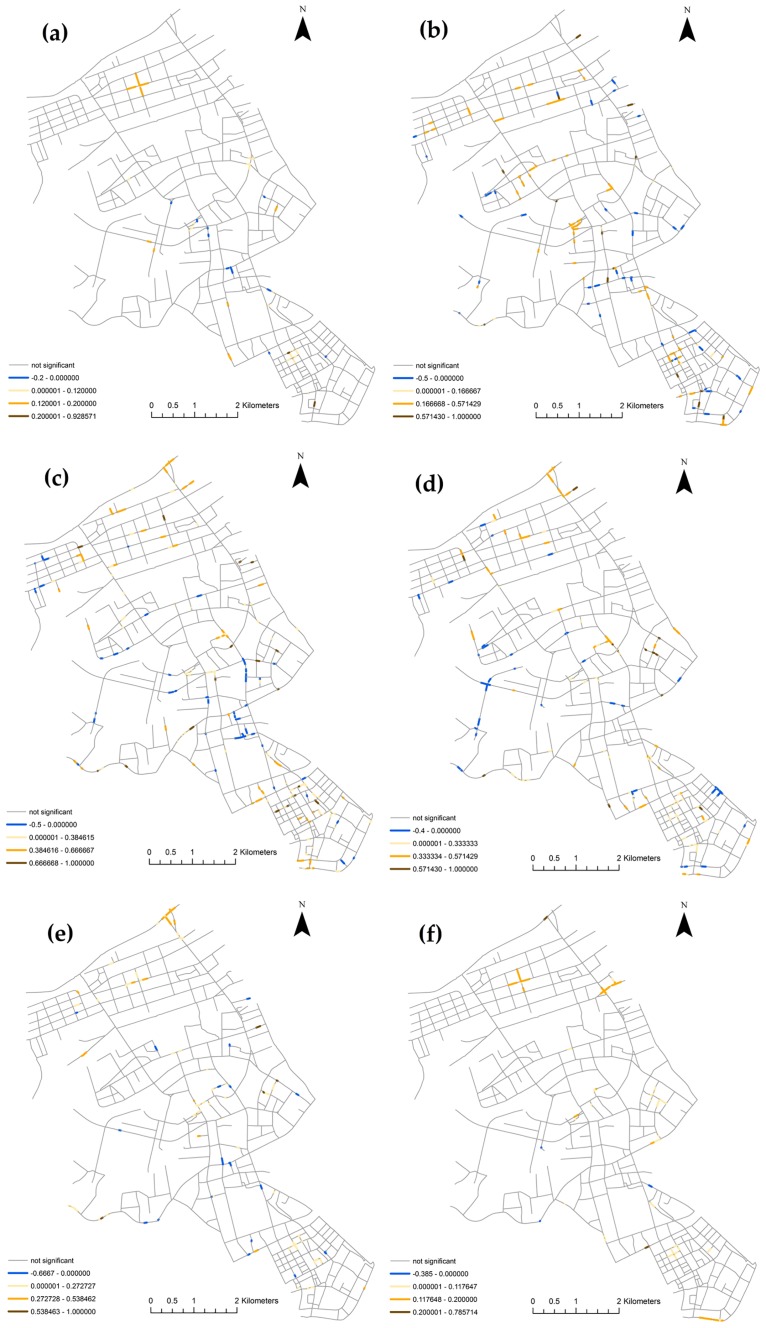
Spatial distribution of the neighbor set LIMA ${\tilde{\tau }}_{i}$ of six consecutive pairs of the six hours per day periods in Jianghan, Wuhan, China: (a) 2–6 to 6–10; (b) 6–10 to 10–14; (c) 10–14 to 14–18; (d) 14–18 to 18–22; (e) 18–22 to 22–2; and (f) 22–2 to 2–6.

**Fig 11:**
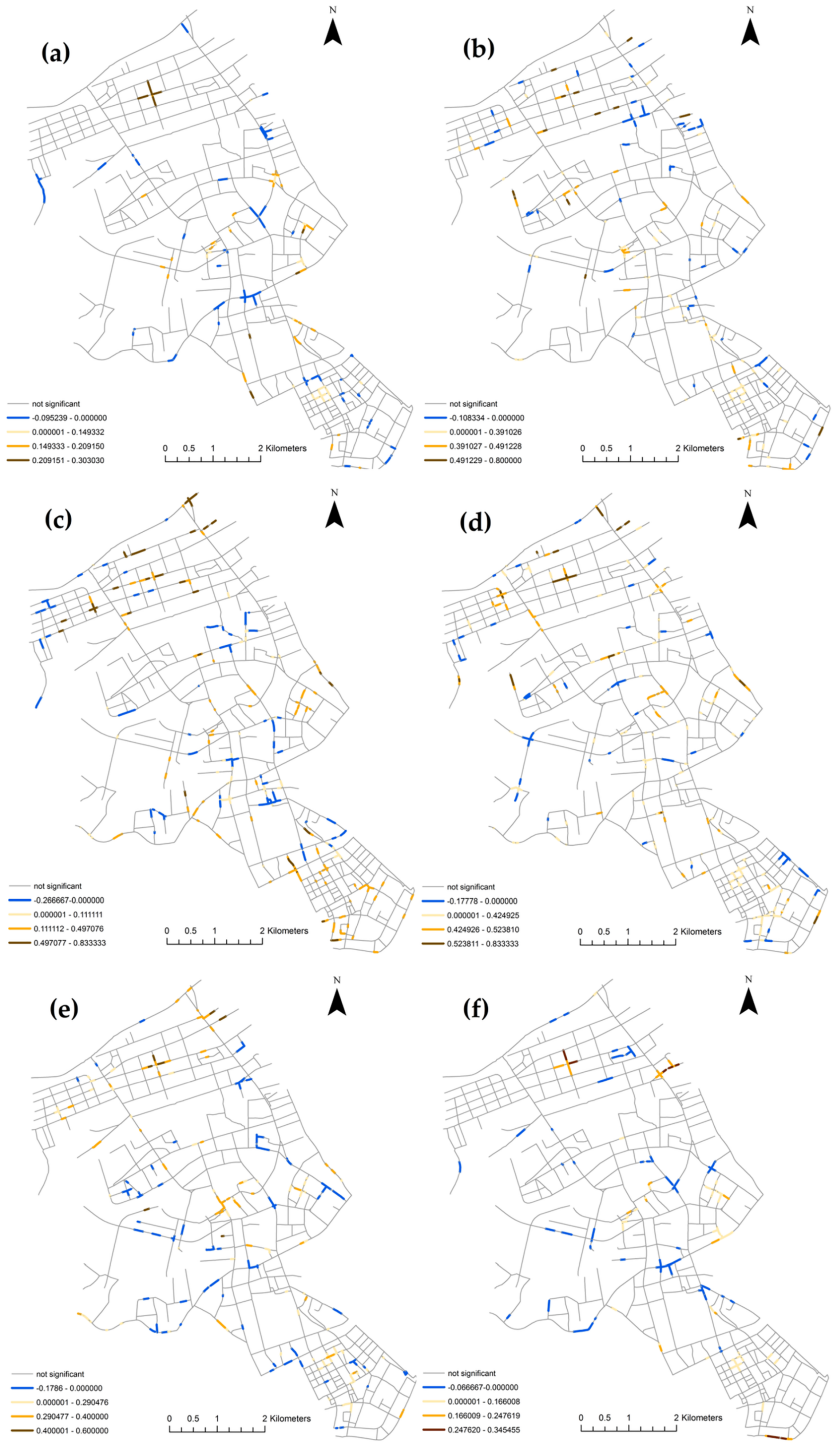
Spatial distribution of the neighborhood set LIMA ${\tilde{\tilde{\tau }}}_{i}$ of six consecutive pairs of the six hours per day periods in Jianghan, Wuhan, China: (a) 2–6 to 6–10; (b) 6–10 to 10–14; (c) 10–14 to 14–18; (d) 14–18 to 18–22; (e) 18–22 to 22–2; and (f) 22–2 to 2–6.

Figs 10 and 11 show the distribution of ${\tilde{\tau }}_{i}$ and ${\tilde{\tilde{\tau }}}_{i}$ values of six consecutive pairs of the six hour of day periods. Although the majority of the roads show a tendency of concordance, the maps show complex local interactions among the segments with significant LIMA measurements. This is reflected by segments with both positive and negative values of ${\tilde{\tau }}_{i}$ and ${\tilde{\tilde{\tau }}}_{i}$ spread throughout the study area. The yellow segments indicate places that tend to retain their ranks among their neighbors or neighborhood set, while the blue segments indicate places that tend to switch ranks with its neighbors or neighborhood set. The results will help practitioners identify roads that are stable in terms of risks, and places with risks that change in certain periods. The maps also capture the most changes in the four period pairs: 6–10 to 10–14, 10–14 to 14–18, 14–18 to 18–22, and 18–22 to 22–2. This is due to the fact that most of the segments have zero collisions during the 22–2 and 2–6 periods. In practice, the network LIMA in this work could be used to identify road segments that have sustained risks across different periods (i.e. the yellow segments), or periodical risks in certain periods (i.e. the blue segments). The periods 6–10, 10–14, 18–22 are periods with vibrant urban activities and largest traffic flows. From 6–10 to 10–14, there are a lot of yellow segments, signaling the traffic patterns are similar from early in the morning until noon. From 10–14 to 14–18, and 14–18 to 18–22, the number of yellow segments has reduced, which indicates more diversified traffic patterns. From 18–22 to 22–2, there are more blue segments than yellow segments, indicating an overall shift of traffic activities. To investigate further the spatial distribution and causes of these sustained or periodical risks at certain road segments, additional data such as the traffic flow, pedestrian traffic, and road characteristics needs to be integrated into the statistical regression analysis.

Compared with the network KDE and network differential Local Moran’s I methods, the network LIMA analysis captures the local interactions of rank changes at the segment level. Figs 10 and 11 convey essentially same information, yet more clusters appear in the ${\tilde{\tilde{\tau }}}_{i}$ distribution visually. However, these clusters are located at roughly the same places as the ${\tilde{\tau }}_{i}$ clusters. In other words, the clusters are more expanded in the ${\tilde{\tilde{\tau }}}_{i}$ maps because all of the neighborhood sets are included in the computations.

## Discussion and conclusions

GIS-based methods have nowadays been used extensively for mapping traffic collisions, which helps practitioners identify hazardous road locations. On the other hand, the demand for understanding the spatio-temporal clustering patterns of traffic collisions, and network-constrained phenomenon in general, have promoted the developments of new models and algorithms within the research community. The advances in handling large datasets empower analysts greatly by providing more effective and efficient ways to integrate multi-source, heterogeneous data. In this context, this work attempted to analyze the spatio-temporal patterns of traffic collisions that integrate the semantic information of the events and surrounding POIs. This work developed a series of network spatial analysis methods for traffic collision analysis. The weighted network KDE method is extended to allow flexible normalization of attribute weights. The differential Moran’s I method and LIMA are extended to the network space in order to detect and quantify the changes of the collision hotspots over time.

The case study proved that these methods could help practitioners to better understand the spatio-temporal patterns and changes in traffic collisions in two ways. First, semantic information is integrated into the analysis methods. Specifically, the weighted network KDE is a simple and straightforward way to integrate the semantics of traffic collisions. The idea of weights could also be embedded in other methods, such as the Knox test, to analyze the spatio-temporal interaction of traffic collisions. The network cross K-function is utilized to analyze the correlation between the dynamic spatial point process of traffic collisions and the fixed spatial locations of POIs. This offers policymakers a new perspective of how collisions interact with the surrounding locations. The second is to analyze data under a meaningful organization of time, such as the hour of the day. This could be done by either conducting analysis at different time points or quantifying the change between time points. The proposed network differential Local Moran’s I and LIMA are such measures to quantify and visualize the hotspot changes over time.

The methods in this work are essentially exploratory, which helps practitioners and policy-makers discover the spatio-temporal clustering patterns from various perspectives at the road segment level. Additionally, the results from these methods are easy to interpret, thus providing a channel for announcing road safety information to the public. These methods could be applied to the analysis of other network-constrained phenomena, such as events of city management. The analysis in this work could be combined with percolation analysis in a traffic network, which offers an innovative perspective to identify bottleneck links considering real-time traffic dynamics [66]. The spatial distribution and patterns of bottleneck links and traffic collisions could be compared to reveal interesting relationships between traffic flow and collisions. Future work will also integrate more data in the analysis pipeline. This includes the attributes related to the roads such as the traffic volumes, road types and weather condition. Furthermore, those attributes related to surrounding POIs could also be integrated, such as the population density, pedestrian traffic, and building structures. These data are also useful for confirmatory analysis in spatio-temporal regressions. In terms of time, the analysis could be done on how the patterns of collisions change around different POIs on certain dates, including holidays, construction, or important events. From a modeling standpoint, the network cross K-function could be extended to the spatio-temporal version for measuring the spatio-temporal aggregations, and it is also critical to analyze the properties of network cross K-function results using proper statistical methods. Additionally, the edge correction issue should be considered in the network space.
